# Surface X-Ray Diffraction Results on the III–V Droplet Heteroepitaxy Growth Process for Quantum Dots: Recent Understanding and Open Questions

**DOI:** 10.3390/s111110624

**Published:** 2011-11-08

**Authors:** Eyal Cohen, Naomi Elfassy, Guy Koplovitz, Shira Yochelis, Sergey Shusterman, Divine P. Kumah, Yizhak Yacoby, Roy Clarke, Yossi Paltiel

**Affiliations:** 1 Applied Physics Department, The Hebrew University, Jerusalem 91904, Israel; E-Mails: naomielfassy@gmail.com (N.E.); guy.koplovitz@mail.huji.ac.il (G.K.); shira.yochelis@yahoo.com (S.Y.); paltiel@cc.huji.ac.il (Y.P.); 2 Solid State Physics, Electro-optics Division, Soreq NRC, Yavne 81800, Israel; E-Mail: sergshus@gmail.com; 3 Applied Physics Program, University of Michigan, 1011 North University Avenue, Ann Arbor, MI 48109, USA; E-Mails: dkumah@umich.edu (D.P.K.); royc@umich.edu (R.C.); 4 Racah Institue of Physics, The Hebrew University, Jerusalem 91904, Israel; E-Mail: yizhak@vms.huji.ac.il

**Keywords:** quantum dots, droplet-heteroepitaxy, surface X-ray diffraction, direct methods, MOVPE

## Abstract

In recent years, epitaxial growth of self-assembled quantum dots has offered a way to incorporate new properties into existing solid state devices. Although the droplet heteroepitaxy method is relatively complex, it is quite relaxed with respect to the material combinations that can be used. This offers great flexibility in the systems that can be achieved. In this paper we review the structure and composition of a number of quantum dot systems grown by the droplet heteroepitaxy method, emphasizing the insights that these experiments provide with respect to the growth process. Detailed structural and composition information has been obtained using surface X-ray diffraction analyzed by the COBRA phase retrieval method. A number of interesting phenomena have been observed: penetration of the dots into the substrate (“nano-drilling”) is often encountered; interdiffusion and intermixing already start when the group III droplets are deposited, and structure and composition may be very different from the one initially intended.

## 1. Introduction

Semiconductor quantum dots (QDs) have drawn a considerable amount of scientific interest since they provide a means to realize “artificial atoms”—zero-dimensional objects in which the charge carriers are confined in all three dimensions. This feature gives rise to potential practical applications of quantum mechanical concepts in the fields of opto-electronics [1,2], quantum information [3,4] and energy harvesting [5–7].

Self-assembly of epitaxial QDs offers a way to fabricate semiconductor sensors with a large density of QDs embedded in the device [8,9]. This facilitates the combination of QD properties with semiconductor device characteristics in a simple, reproducible way, without the need for costly and time-consuming lithography steps [10,11]. Fabrication of self-assembled QDs via the Stranski-Krastanov (SK) growth mode is widespread and has been extensively studied [12–15]. The formation of three-dimensional islands in the SK method is driven by strain requiring a large lattice mismatch between the substrate and dot materials.

An alternative, more recent technique to grow QDs is the droplet heteroepitaxy (DHE) method [16]. This method consists of two basic steps: first, liquid phase nano-droplets of group III elements are formed on the substrate. Subsequently, these droplets are exposed to a gas flow of one or more group V elements. Although the growth process starts with liquid droplets, at the end, under proper conditions, one ends up with single crystal dots, atomically registered with the underlying substrate. The method is known to be very sensitive to growth conditions, yet it is not constrained, as is the SK method, to materials with a large substrate-dot lattice mismatch. A variety of highly crystalline and high density nanostructures, grown on various substrates have already been demonstrated using the DHE method. These include combinations of GaAs, GaN, InGaAs, InAs, InSb, InAsSb and GaSb [17–21] Interestingly enough, in some of those systems, the dots were realized with practically no substrate-dot lattice mismatch [17,21]. Complex shape control has also been achieved by the droplet method [22].

These systems demonstrated quantum behavior in several devices. However, the same feature of quantum confinement responsible for the interest in QDs also limits our ability to fully exploit their potential for applications. Quantum confinement in QDs, and their electronic and optical properties, depend strongly on size, shape, chemical composition and strain-fields in the dots. Since self-assembly is a statistical process, these properties often have a fairly broad distribution. Moreover, the DHE growth process is rather complex and involves many growth control parameters. It is therefore of great importance to understand the exact mechanisms participating in the growth process. Many studies have been made in order to characterize the structural and compositional parameters of QDs grown by the SK method [11,23]. At present, knowledge of the DHE systems is more limited and in both systems precise structural information at the atomic level and understanding of the mechanisms taking place in the QDs growth process is yet to be achieved.

Many characterization methods are used for the study of nanostructures. These include direct mapping methods such as AFM, TEM, STM and cross-sectional STM [11,23], as well as indirect band structure studies such as optical measurements [11]. Diffraction of X-rays offers another powerful tool to investigate the dot structure with sub-angstrom resolution. The main advantage of X-ray diffraction (XRD) is the fact it is non-destructive, yielding information about the structure without altering the sample properties during its measurement or preparation. In addition, the ability of X-rays to penetrate into the dots and the substrate makes it possible to investigate both surface and buried structures. However, this property of X-rays in turn makes the scattered signal relatively small, requiring grazing incidence-angle geometry measurements and the use of bright synchrotron radiation sources. XRD measurements yield the diffraction intensities along Bragg rods in reciprocal space. Reconstruction of the real space electron density from these measurements is generally not straightforward. To obtain the real space electron density it is necessary to use either complex modeling and fitting or direct methods.

## 2. Experimental Section

### 2.1. Fabrication of Quantum Dots by Droplet-Heteroepitaxy

**Figure 1.**
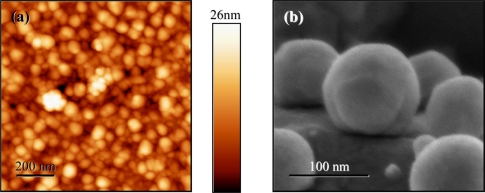
High-resolution AFM and SEM images of QDs grown by DHE. (**a**) AFM image of InAs/GaSb QDs. Average height of dots is ∼11.5 nm above surrounding surface, with a fairly large distribution. Places where several droplets have merged to form particularly large dots are also visible. (**b**) Tilt-geometry high-resolution SEM scan image of InSb/GaAs dots, visualizing the non-wetting, obtuse contact-angle nature of the dots. The dots shown here are larger than those measured in the XRD experiment.

In this paper we review the results of surface XRD measurements of several III–V QD systems grown by the DHE method [24,25]. Complementary measurement techniques are presented as well. The samples were grown in a metalorganic vapor phase epitaxy (MOVPE) reactor. For the growth, we have used epitaxial-ready GaAs or GaSb substrates, nominally (001) oriented, heated to 370–390 °C. Liquid In droplets were then formed by supplying a 2 s flow of 150 cc/min trimethylindium (TMIn) at a substrate rotation speed of 200 rpm. After cooling down to 330–350 °C, the droplets were exposed to either 120 cc/min of tertiarybutylarsine (TBA) or 45 cc/min of trimethylantimony (TMSb) for 15 s—to form InAs or InSb nano-structures, respectively. A detailed description of the growth process is described elsewhere [26]. Figure 1 shows representative high-resolution SEM and AFM images of some of the samples. From such micrographs, the density of dots was estimated and found to be higher than 10^11^ dots/cm^2^, for all samples investigated.

### 2.2. Direct Surface X-Ray Diffraction Measurements and Analysis

Surface XRD measurements were carried out at beam lines 7-ID and 33-ID of the Advanced Photon Source (APS), Argonne National Laboratory. We used a direct phasing technique known as coherent Bragg rod analysis (COBRA) [27–29] to analyze the results. COBRA is able to solve the structure of systems with two-dimensional periodicity, such as epitaxial thin films, and can also be applied to the case of epitaxial QDs [24,30]. A detailed description of how COBRA works, and of the assumptions behind it, is given in ref. [27]. Briefly, in this method, diffraction intensities are measured along substrate-defined Bragg rods. The data is then analyzed using COBRA in order to retrieve the diffraction phase information. Using the diffraction intensities and phases, a set of complex scattering factors is obtained for a number of symmetry inequivalent Bragg rods. This set of complex scattering factors is then Fourier transformed into real space yielding a three-dimensional electron density map of the ‘folded’ structure. The folded structure is the structure obtained by laterally translating each atom in the system into a substrate-defined 2D unit cell, using the substrate-defined unit cell vectors in the two lateral dimensions [27]. Lateral information on the dots structure could be obtained using the ‘folded structure’. Assuming that the center of the dot is lattice matched to the substrate, the electron density peaks of this part will be registered with the substrate periodicity. Strain layers which fit the outer shell of the dots will form side peaks. Due to the X-ray spot size, diffraction intensities are averaged over a 1 mm × 1 mm area.

COBRA also exploits anomalous scattering by collecting diffraction data for each rod at different beam energies. By working at two beam energies—near and away from the absorption edge of an element in the structure—a difference in the element’s scattering factors can be achieved. This can then be used to obtain the relative concentration of the element in the folded structure [29]. For all samples under investigation, we recorded intensities along Bragg rods at two beam energies: at E_1_ = 10.362 keV—just below the Ga K-Edge, and at E_2_ = 11.864 keV—just below the As K-Edge. The Ga scattering cross section at energy E_1_, *f*_*Ga*1_, is lower by a factor of about 2/3 than its cross section at E_2_, *f*_*Ga*2_, and vice versa for the As scattering cross sections, *f*_*As*1_ and *f*_*As*2_ (*f*_*As*1_ is approximately 3/2 of *f*_*As*2_). The cross-sections of the other elements remain essentially unchanged. So, for example, at sites of group III atoms in the common zincblende lattice structure of III–V alloys, the ratio between Ga and In, *R_Ga:In_*, is given by:

(1a) $$R_{\mathrm{Ga}:\mathrm{In}}=\frac{f_{\mathrm{In}}-\eta_{\mathrm{III}}f_{\mathrm{In}}}{\eta_{\mathrm{III}}f_{\mathrm{Ga}2}-f_{\mathrm{Ga}1}}$$

Similarly, at group V sites, the ratio between As and Sb, *R_As:Sb_*, (in systems where Sb is present) is given by:

(1b) $$R_{\mathrm{As}:\mathrm{Sb}}=\frac{f_{\mathrm{Sb}}-\eta_{V}f_{\mathrm{Sb}}}{\eta_{V}f_{\mathrm{As}2}-f_{\mathrm{As}1}}$$

where *η_III_* / *η_V_* is the ratio between the effective electron charge measured at the two energies at either group III or group V atomic locations. This kind of calculation is carried out for each peak in the electron densities maps to yield the ratio of the respective elements’ concentration at that site as a function of distance from the interface.

Another advantage of COBRA is its extraordinary speed, in analyzing the measured XRD data, owing to the direct phasing step. Only a few tens of iterations are usually needed to converge to a reasonable agreement between the measured results and the diffraction intensities calculated from the electron density determined by the COBRA phasing technique.

## 3. Results and Discussion

Three different DHE-grown QD systems are used here to illustrate the measurement of QDs and the COBRA direct phase retrieval analysis approach: InAs/GaAs [24], InSb/GaAs [25] and InAs/GaSb. InAs/GaAs is a relatively simple system—being composed of only three elements, and the SK-grown equivalent system has been widely studied [12–15], and also analyzed by COBRA [30]. InAs and GaSb are two materials with less than 1% difference in their lattice constant, and so the InAs/GaSb is an interesting system which can be grown via the DHE method, but not by SK. In order to better understand the growth processes, two samples—at two different stages of the growth process were investigated for this system: first, with only In droplets on the substrate and second, with the process complete after the exposure of those droplets to As flow.

**Figure 2.**
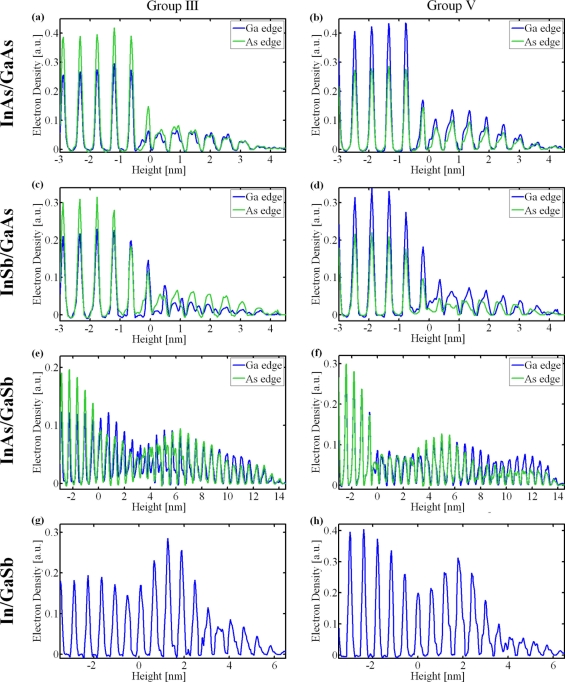
Electron density profiles of the different QDs systems; InAs/GaAs, InSb/GaAs, InAs/GaSb, In/GaSb. For each, a profile line passing through the group III elements (left) and the group V elements (right) are displayed. Line profiles are plotted for two different beam energies used: below the Ga K-edge and below the As K-edge.

Figure 2 shows the electron density profiles of the three systems. The graphs show the electron density along lines going through the centers of group III and group V sites in the III–V zincblende unit cell structure of the lattice, along the (001) direction, perpendicular to the surface. Two graphs are displayed for each line—corresponding to X-ray beam energies just below the Ga and As K-edges. The last two plots showing dots grown on the GaSb substrate represent preliminary results with errors about four times larger than usual. These results are therefore considered only qualitatively, namely there is order in the In/GaSb system close to the interface, and that the dots of the InAs/GaSb shows many more layers than the In/GaSb.

**Figure 3.**
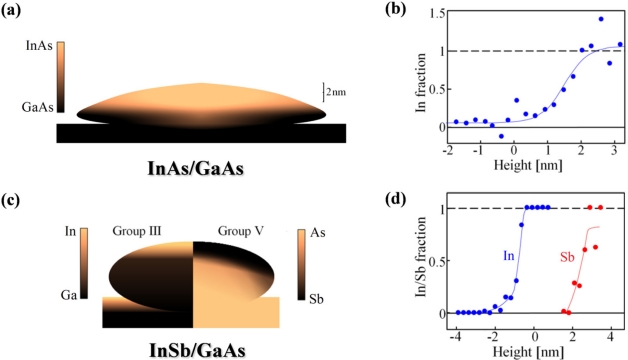
Compositional schemes of the different dot systems. (**a**) Shape and chemical composition map of InAs/GaAs QD. (**b**) Average fractional occupancy of In in the In_x_Ga_1-x_As structure as a function of height from the dot-substrate interface. (**c**) Composition map of InSb/GaAs QD. The left half shows the group III (In/Ga) elements occupancy, and the right half—that of group V (As/Sb). (**d**) Average fractional occupancy of In and Sb in the In_x_Ga_1-x_As_y_Sb_1-y_ structure as a function of height from the dot-substrate interface.

In the first two samples, the electron density peaks of the dots (above the nominal interface position, at height = 0) are non-Gaussian and broader than the narrow Gaussian-like peaks of the substrate. The overall shape of the dots can be inferred from the electron density height distribution and is in agreement with the shape deduced from AFM and HRSEM measurements, excluding a thin oxidized layer at the outer shell of the dot—visible by AFM, but not by the XRD profile, and is shown schematically in Figure 3a. In both samples an obtuse, non-wetting contact angle between the dots and the substrate can be identified. This can be seen by tilt-geometry high-resolution SEM images (see Figure 1b), This feature was also observed in the SK grown InAs/GaAs dots [30].

In both systems the dots extend a few mono-layers below the substrate-dot interface. A related phenomenon has been observed earlier in related systems where In and Ga have been observed to “nano-drill” into a III–V substrate [31]. It seems that the effect of nano-drilling is more pronounced in the InSb/GaAs system. This could be a result of the fact that this system was grown at a 20 °C higher temperature than the InAs/GaAs system. However it could also be a result of the presence of Sb that acts like a surfactant speeding and intensifying both nano-drilling and intermixing effects [32,33].

The profile of the electron density peaks in both systems has a quasi-rectangular shape convoluted with a Gaussian (seen most clearly at the InAs/GaAs system). This is ascribed to the bowing of the atomic layers in the dots, because their lattice constant is larger than that of the GaAs. This feature was also observed in QDs grown by Stranski-Krastanow method [30]. In both systems, the dots’ electron density peaks seem to have a small dip at the center. This could be a result of edge dislocations at the interface between the substrate and the dot. Such dislocations have been observed in DHE-grown dots by cross-section TEM scans [34], and might take part in relieving strains caused by the lattice mismatch. Folding a three-dimensional structure containing strains and dislocations in such dots [35], yields split electron density peaks very similar to those observed in our measurements [24].

We now focus on the chemical composition mapping of the dots, made possible by the analysis of two different X-ray beam energies. As described earlier, we can determine the In/Ga and As/Sb ratios in each atomic layer, thereby enabling us to map the chemical composition as a function of the height from the interface. In both systems, intermixing of materials from the substrate is observed, and the resulting structure is somewhat different from the nominal one. The compositional maps are displayed in Figure 3.

We will start with the simplest system studied—InAs/GaAs (see Figure 3a). In this system diffusion of elements from the substrate into the dots was observed, forming a layer of InGaAs with gradual decrease of the Ga fraction towards the top of the dots (see Figure 3b). Within the experimental accuracy, at a height of approximately 2.3 nm from the substrate-dot interface and above, only InAs is present. TEM characterization indicates that in some systems, the Ga diffuses further up at the dot’s perimeter than at the center [36]. Comparison of these results with those obtained on InAs/GaAs dots grown by the Stranski-Krastanov method [30], reveals that the Ga diffusion is somewhat larger in the DHE grown dots. However, since temperature greatly affects the inter-diffusion process, the exact difference may depend on specific growth parameters.

The structure of the InSb/GaAs sample turned out to be quite surprising. The COBRA results show that the top 2–3 mono-layers of the substrate are InAs. This is evident from the fact that in these layers the effective electron densities measured using the two energies are essentially equal (see Figure 2c) [25]. The center part of the dot consists mainly of GaAs while the outer shell consists mainly of GaSb [25]. This ‘core-shell’ structure is represented schematically in Figure 3c and the layer-by-layer In concentration in the substrate and Sb concentration in the dots are shown in Figure 3d. The final shape of the dots matches the shape seen by high resolution TEM on a capped dot system [37].

The results of the preliminary XRD measurements on the InAs/GaSb and In/GaSb samples have been analyzed with COBRA and show some very interesting results. The In in the In/GaSb sample was deposited under exactly the same conditions as in the InAs/GaSb sample. In is a polycrystalline metal. Consequently the positions of the In atoms are not correlated with those of the substrate. So, the electron density in the In/GaSb folded structure is constant and cannot be seen by COBRA. The results seen in Figure 2g and 2h show electron density peaks extending to about 5 nm above the substrate-dot nominal interface. This means that within this distance the In has already reacted at least partially with group V elements. We do not know at this point whether it reacted with Sb from the substrate or with As from the vapor used to treat the substrate, or both. Further measurements at X-ray photon energies just below the Ga and As edges are needed to answer these questions. Notice that the average dot height in the InAs/GaSb samples is more than double that of the In/GaSb dots (5 nm). This is indeed what we expect. Namely that after the In droplets are exposed to As, the liquid In metal that did not react in the first stage reacts with As, and crystallizes epitaxially with the substrate. It is reasonable to speculate that both stages of growth can be described as a nano Liquid Phase Epitaxy; the liquid In melts back the substrate and retains a saturated solution in the vicinity of the substrate surface. This saturated solution becomes super saturated when atoms from the gas phase diffuse or when the cooling down stage starts creating “nano liquid epitaxial growth”.

In contrast to the broadened electron density peaks of the first two samples, the electron density peaks in Figure 2e–2h are rather sharp and Gaussian-like. This means that the mono-layers in the dots are flat, probably because the InAs/GaSb system is almost strain free.

**Figure 4.**
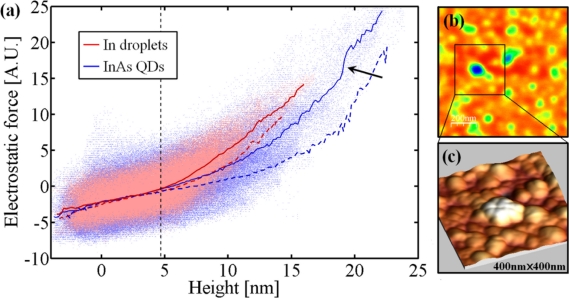
(**a**) Scatter plot of EFM signal *vs*. height for In droplets (pink) and InAs QDs (light blue) on GaSb substrate. The solid lines represent the average at a given height. The dashed lines are the averages obtained for measurements with a positive tip voltage (scatter plot not shown). The sudden “jump” marked by the arrow, occurs due to points corresponding to the area highlighted in the EFM scan image shown in (**b**). A closer look at the topography of the area is shown in (**c**).

Electrostatic Force Microscopy (EFM) measurements [38], comparing the In/GaSb and InAs/GaSb samples support the presence of a crystalline III–V compound at the base of the In droplets. A 1 μm × 1 μm area has been scanned for topographic height and electrostatic force. The electrostatic force sensed by the tip increases with the carrier density in the material underneath, and decreases with the distance of the tip from the surface. A plot of the electrostatic force *versus* height measured by AFM is presented in Figure 4a while the EFM and AFM maps are shown in Figure 4b and 4c, respectively. The large scatter at each height is a result of the fact that the lateral resolution of the EFM signal is about 20–30 nm while the height resolution of the AFM measurement is better than 1 nm. Thus at different points with the same height the surrounding landscape may be very different yielding different EFM signals. Several 1μm x 1μm areas were measured with different topographic structure with both negative and positive tip voltages, all showing the same trends.

The average electrostatic force as a function of height for InAs/GaSb and In/GaSb at both positive and negative tip voltages are also shown in Figure 4a. Note that the force on the negatively biased tip is larger than the positive one. This indicates that the InAs dots are p type. At heights below 5 nm the average force in both samples is approximately equal while at larger heights the forces in the In/GaSb sample are significantly larger than those in InAs/GaSb. This suggests that up to a height of about 5 nm the In has reacted with group V elements converting the metal into a semiconductor while above it the dots are more and more metallic. This supports the COBRA results. Finally, note that in the InAs/GaSb sample, there is a small jump in the electrostatic force at about 19 nm (indicated by the arrow). This corresponds to the large peak surrounded by a square in Figure 4b and indicated by a light color in Figure 4c. This jump suggests that the top of this peak is still metallic and not enough As was supplied to convert the tops of the large peaks into a semiconducting state.

The results obtained so far suggest some insights with respect to the DHE growth process. The fact that the bottom parts of the In droplets convert into a crystalline semiconductor before they are exposed to group V vapor suggest that the liquid In interacts chemically with the substrate. This suggests that nano drilling and inter-diffusion may be taking place already at this stage. It is also possible that the replacement of Ga by In at the top mono-layers of the substrate observed in InSb/GaAs takes place at this stage.

During the second stage of the growth process, As or Sb from the metalorganic source diffuses into the liquid In. The interface composition depends on the growth conditions and the materials involved. For example in the InSb/GaAs sample, Sb acts as a surfactant and concentrates at the outer shell of the dots while As concentrates inside the dot. The crystallization process proceeds from the substrate upwards as in liquid phase epitaxy. This is supported by the fact that the tops of large peaks remain metallic when the supply of As is insufficient.

**Figure 5.**
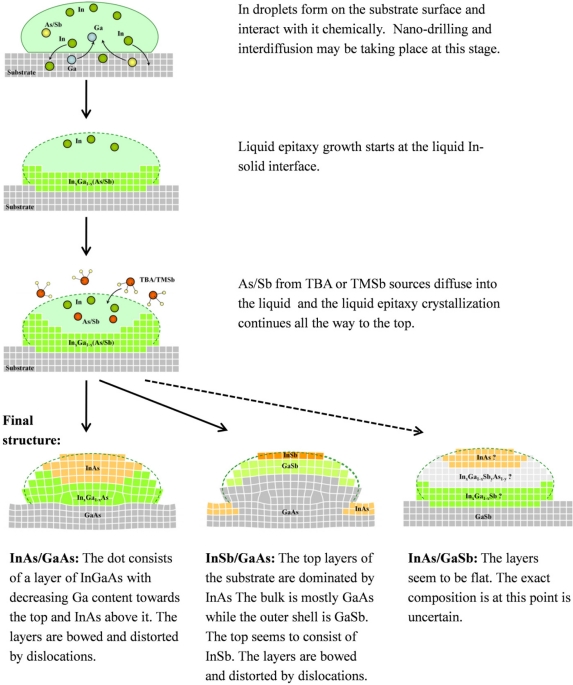
Schematic illustration of the suggested model of the DHE growth process. The final structure is different for the three different systems investigated.

Strain affects the shape of the monolayers. When the mismatch between the dots and the substrate is large, the atomic planes in the dots are bowed but they are flat when the mismatch is small. Figure 5 illustrates the above insights schematically.

Further investigation is needed to answer a number of questions:

1. The results show that the In droplets interact chemically with group V elements at the earliest stage. However, it is not clear whether it interacts with the As used to prepare the substrate surface or with the substrate group V element; and if so, what happens to the remaining group III element?
2. The InAs/GaSb system is very complicated and the dots we studied were quite large. The chemical composition of smaller dots needs to be further investigated.
3. We do not know at present at what stage the bowing of the layers takes place.
4. The effects of adding an epitaxial cap layer on top of the QDs is of major importance and should be carefully investigated.
5. Other material combinations should be investigated at different growth stages before some more general conclusions can be firmly established.

## 4. Conclusions

We have applied a direct phasing method for the analysis of surface X-ray diffraction measurements of a set of QDs fabricated by the DHE technique. The results provide three-dimensional electronic density maps of the dots’ folded structure, including information on their chemical composition. The results indicate that quite often the final structure is considerably different from the nominal one, with high sensitivity to growth conditions and the materials involved. A number of interesting phenomena have been observed. In some of the systems, we have observed that the dots nano-drill into the substrate. In all the samples we have studied, we have observed inter-diffusion of both group III and group V elements. In one case, we observed that the In from the droplet replaces the Ga at the top substrate mono-layers and the Sb concentrates in the outer shell of the dots while the dot center contains mainly As that has diffused from the substrate. We have seen that in samples with large dot-substrate mismatch the layers are bowed, but are flat when the two are matched. Finally the liquid In droplets interact chemically with the substrate already in the first growth stage and some of the above phenomena may be taking place already at this stage. These results tell us that it is very important to investigate the atomic structure and composition of quantum dots systems because they may be quite different from the structure and composition intended by the grower.
